# Definitions of ultra-processed foods beyond NOVA: a systematic review and evaluation

**DOI:** 10.29219/fnr.v69.12217

**Published:** 2025-06-16

**Authors:** Anine Christine Medin, Stine Rambekk Gulowsen, Synne Groufh-Jacobsen, Ingunn Berget, Ida Synnøve Grini, Paula Varela

**Affiliations:** 1 Kristiansand Norway, Department of Nutrition and Public Health, Faculty of Health and Sport Sciences, University of Agder, Priority Research Centre Lifecourse Nutrition, Kristiansand, Norway; 2 Nofima AS, Ås, Norway

**Keywords:** processed foods, highly processed foods, classification system, food categorization, food processing, nutrition, diet

## Abstract

**Background:**

Ultra-processed foods (UPFs) are associated with negative health outcomes, but current classification systems, including the dominant NOVA system, are typically not suitable for identifying which factors of these foods may be harmful. New ways of defining UPFs are needed to better understand how food processing affects health.

**Objective:**

To identify classification systems that include a category for ultra-processed or highly processed foods with a focus on comparing their definitions and provide a current evaluation of available alternatives to NOVA.

**Design:**

A systematic literature review was conducted following the Preferred Reporting Items for Systematic reviews and Meta-Analyses (PRISMA) guidelines, with the search strategy developed in collaboration with a university librarian. The literature search was completed on 18 December 2023, using databases Medline, Embase (via Ovid), and Web of Science. No human participants were included.

**Results:**

We identified six systems – NOVA, European Prospective Investigation into Cancer and Nutrition (EPIC), International Food Policy Research Institute (IFPRI), University of North Carolina (UNC), UnProcessed Pantry Project (UP3), and Siga – that categorize highly processed food or UPFs. These systems differ in structure and detail, with NOVA, EPIC, and Siga providing specific examples of processing techniques. Regarding additives, NOVA, Siga, and UP3 include them explicitly, with Siga offering the most detailed categorization based on additives and ingredients. Siga also includes quantitative measures for nutritional quality, including cut-offs for sugar, fat, and salt, while IFPRI and UP3 address nutritional quality non-quantitatively.

**Discussion:**

When comparing NOVA’s UPF category with the highly processed food or UPF categories used in the other five identified systems, we found that none specifies processing techniques clearly. Both NOVA and Siga define additives unique to their UPF categories. Siga stands out by addressing the diverse risks associated with additives and offering quantitative nutritional quality criteria, thus addressing some of the criticisms of how UPFs are commonly defined.

**Conclusions:**

Siga represents a valuable, but not final, step forward in classifying UPFs and could serve as a reference in developing a new operational definition for UPFs.

## Popular scientific summary

- Current food classification systems based on food processing, especially the popular NOVA system, have been criticized for not defining ultra-processed foods in a useful way.
- This systematic review identified six systems: NOVA, EPIC, IFPRI, UNC, UP3, and Siga.
- When comparing their definitions for better alternatives, Siga stands out by distinguishing between additive types and including quantitative measures for nutritional quality. Siga could be a step forward in developing a new operational definition for ultra-processed foods.

Since Monteiro introduced his NOVA food classification system in May 2009 – focusing on the extent and purpose of food processing rather than food groups or nutrients (1–3) – the term ultra-processed foods (UPFs) has gained recognition along with a growing research on their negative associations with health. According to the NOVA system, UPFs are foods made from ingredients not typically used in a home kitchen that undergo a series of industrial processes (4).

Over the last six decades, considerable advancements in food science and retailing have shifted population diets toward consumption of UPFs (5). This shift seems to have reached a point of saturation in the global north, while the consumption of UPFs continues to rise in the global south. This is evident from the volume sales data of UPFs worldwide from 2002 to 2016, which shows an increase in UPF sales except in Western Europe, North America, and Australasia (6). Conservative estimates from UPFs consumption data across 22 European countries show energy intakes from UPFs between 14 and 44%, with the highest intakes in the Netherlands, Germany, the UK, and Sweden (7). Reports from Australia and the US show that UPFs contribute with 42 and 58% of energy intake, respectively (8, 9).

Numerous studies examining the associations between exposure to UPFs, as defined by the NOVA food classification system and adverse health outcomes, have been published over the last years. In 2024, a comprehensive umbrella review including 45 meta-analyses and ~10 million study participants shows that there is convincing evidence for higher risk of cardiovascular disease-related mortality, type 2 diabetes, anxiety, and other common mental disorders with an increased total exposure to UPFs (10). It has been suggested that the explanation for these findings may be due to the average poor nutrient profiles of UPFs, which is supported by a meta-analysis from 13 countries showing that a high intake of these foods is associated with low nutritional quality (11).

However, recent studies have identified varying associations between UPFs and health, when comparing risk of total versus subgroup consumption of UPFs. In 2023, Chen et al. showed that total UPF intake is associated with a higher risk of type 2 diabetes, driven by subgroups like white bread, soft-drinks, sauces, animal-products, and spreads, whereas ultra-processed wholegrain bread and yogurt- and dairy-based desserts, fruit-based products, and some snacks were associated with a lower risk (12). Cordova et al. showed an increased risk of multimorbidity of cancer and cardiometabolic diseases with higher intakes of total UPF, but not with ultra-processed bread, cereals, or plant-based alternatives (13). Moreover, a recent 2024 paper found that the association between higher total intake of UPFs and increased risk of cardiovascular disease was largely driven by processed meat and soft drinks, and that ultra-processed bread, cold cereals, yogurt, dairy-desserts, and savory snacks were associated with reduced risk (14). However, a large review of 37 cohort studies suggests that the observed negative association between UPFs and health outcomes cannot be explained by poor diet quality alone (15), but it remains unclear to what extent the negative health outcomes associated with UPFs consumption are driven by their processing.

The mechanistic explanations for the observed associations between UPFs and adverse health outcomes are currently uncertain, but proposed factors that need to be explored further include UPFs’ high average energy density and nutrient-poor composition, alterations in their food matrix, the presence of harmful compounds from processing or packaging, and their content of food additives (16). Unfortunately, the current classification systems, including the dominant NOVA system, are typically not designed to identify the specific food processes, nutrients, or non-nutrient-based factors associated with UPFs that may be harmful. Critics of NOVA argue that its definitions lack scientific, measurable criteria (17), leading to confusion and misclassification among both consumers and researchers (18, 19). Additionally, it has been argued that it is premature and wrong to implement any public policies recommending limiting or avoiding UPFs as defined by NOVA (20, 21).

Given the growing evidence linking UPFs consumption to adverse health outcomes and the limitations of NOVA used in most of these studies, there is a need for alternative ways of classifying foods based on their processing. An early systematic review by Monteiro’s group (22) and a critical literature review by Sadler et al. (23) evaluated existing systems based on food processing at that time. The review by Monteiro’s group in 2014 identified and ranked several early systems, concluding that the first NOVA version, available at that time, was the best, despite being described as ‘mostly clear’ and ‘mostly workable’. However, this review did not account for later criticisms of NOVA. The more recent 2021 review by Sadler et al. identified additional food processing classification systems focusing on purpose and place of processing in addition to extend and nature of change of the processed foods, but without using the rigorous approach in a systematic review. They conclude that greater clarity is needed about the role of additives and the food matrix in processed foods and their effects on health. Although several narrative reviews and comparison studies of food processing classification systems have been conducted over the last years, there has not been conducted a thorough systematic review on food-processing classifications systems focusing on further details in the highly processed/UPFs definitions as compared to NOVA category four.

The substantial increase in UPFs-related publications suggests a need for an updated review. This study aims to address this by first conducting a systematic review to identify all existing classification systems having a UPF or highly processed food category and comparing their definitions to provide a current evaluation of the available alternatives to NOVA.

## Methods

### Search strategy and eligibility criteria

This study adhered to the Preferred Reporting Items for Systematic reviews and Meta-Analyses (PRISMA) guidelines (24) where applicable; adaptations were made as quantitative data extraction was not within the scope of this paper. The search strategy was developed by the authors in collaboration with a specialist university librarian. The literature search was conducted on 18 December 2023, in Medline, Embase (via Ovid), and Web of Science (WoS). The search terms used are given in Table 1 and include combinations of word lemmas for classification, ultra-processed, or high-processed, near food or diet.

**Table 1:** Search terms used in the different databases

Database	Search terms
Ovid	(((Classify* or classification*) and (Ultraprocess* or ultra-process* or high* adjd6 process*)) adj3 (food or diet*))
WoS	TS=(classify* OR classification*) AND TS=((Ultraprocess* OR ultra-process* OR (high* NEAR/5 process*)) NEAR/2 (food OR diet*)) AND PY=2009–2024

adj6: Searches for words that are at most six words apart; adj3: Searches for words that are at most three words apart; NEAR/5: Searches for words that are at most five words apart; NEAR/2: Searches for words that are at most two words apart; PY: Publication year.

Articles were included if they described one or more classification systems that defined foods and beverages as highly or ultra-processed in at least one category. Articles were excluded if they were not published in English, were not peer-reviewed, or were published before 2009, and the time classification systems based on processing started to proliferate. Finally, articles that did not describe an original classification system, or an updated version of an original classification system for the first time, were excluded as a final step.

### Selection of articles

The literature search was processed through Rayyan.ai for screening using blind mode (25). Screening was conducted independently by SRG, SG-J, and ACM. In the first step, SRG reviewed all abstracts, while SG-J and ACM each reviewed half of the abstracts. All abstracts were assessed based on the predetermined inclusion and exclusion criteria. At this stage, percentage agreement between reviewers was 98%, as calculated in Rayyan. In the second step, discrepancies in evaluations were resolved through discussion after reviewing the full texts, with the authors collectively deciding on the inclusion or exclusion of each study. In the third step, before the final exclusion of articles that did not describe original systems or updates, a reference check of all included articles was conducted. This step aimed to identify additional relevant articles that met the same inclusion and exclusion criteria applied during the initial search.

### Data extraction

Data relevant for extraction were discussed and jointly determined by SRG, SG-J, and ACM. Following this, the data were extracted from the articles by SRG and then checked by ACM. The data extracted from selected articles included: author(s), publication year, geographical study setting (or if not applicable, first author’s country of affiliation), study type and design, study objective(s), name of the classification system(s), and whether the study introduced the system for the first time, described an updated version, or referred to an existing system. Additional details of the classification system’s structure were extracted, including number of categories, a brief description of each category, and a detailed description of the definitions of the UPFs or highly processed food category. Further data specific to definitions of the UPFs or highly processed food category were also extracted, including whether specific types of food processing were described, whether additives were included, and whether nutritional quality was included.

## Results

**Fig. 1:**
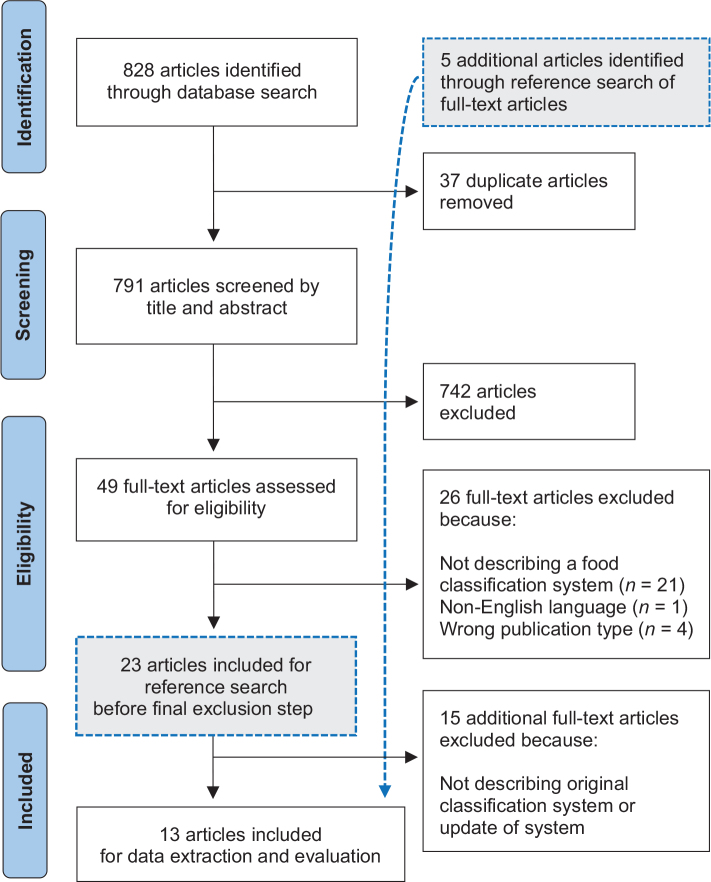
Flow chart of the selection of articles.

The literature search resulted in the inclusion of 13 articles describing original classification systems or updated versions of these. Of these, eight were identified through the main search, and five additional articles were identified through references searches (as described in the third step of selection of articles). The five additional articles were found within the 23 full-text articles, which fulfilled inclusion and exclusion criteria before the final exclusion step. The full study selection process is detailed in the PRISMA flow chart (Figure 1).

The main characteristics of the 15 articles excluded at the final exclusion step are summarized in Supplemental Table S1. Six of these articles were various forms of reviews of existing classification systems (21–23, 26–28), three were comparative studies of existing classification systems (29–31), and the remaining three were miscellaneous studies describing existing classification systems or their use (18, 32–36). From these excluded articles, four classification systems based on food processing were identified but did not meet the criteria for inclusion in this review. These included one Brazilian system by Louzada et al. (37), which was not considered sufficiently distinct from the NOVA system, and three others lacking defined categories for UPF/highly processed foods: the National Institute of Public Health in Mexico (NIPH) system from 2007, described in the review by Moubarac et al. (22); the International Food Information Council (IFIC) system, first applied in 2012 (38); and the Food Standards Australian New-Zealand (FSANZ) system, described in the review from Jideani et al. (27).

### Characteristics of the included articles

An overview of the 13 included articles is shown in Table 2, with publication years spanning from 2009 to 2021. A total of seven articles are introducing and describing a food processing classification system with a category for ultra-processed or highly processed foods for the first time (1, 37, 39–43). These were as follows: The NOVA system by Monteiro et al., first introduced in 2009 (1) and later described in more detail (2); a un-named system by the European Prospective Investigation into Cancer and Nutrition (EPIC) in 2009 (39); a system from the International Food Policy Research Institute (IFPRI) (40); a system from the University of North Carolina (UNC) (41); one from the UnProcessed Pantry Project (UP3) (42); and finally, the Siga system from 2020 (43). Six articles describe later updates of four of these systems, namely, the updated NOVA in 2016 (3) with additional detailed descriptions in 2017 (4) and 2019 (44), the updated EPIC system in 2011 (45), an update of UNC in 2020 (46), and a new version of the Siga in 2021 (47). Seven of the identified articles are from the global North, with four from Europe and three from the United States. The remaining six articles are from South America, five of which describe the NOVA classification system.

**Table 2:** Main characteristics of included articles ordered chronologically by group^a^

Authors, year	Study setting^b^	Group^a^, Study type	Objective(s)/*description of classification system(s) in study if not clear from objective(s)*	System, *version*
**Monteiro et al. 2009 (** **1** **)**	Brazil	(i), Commentary	*Proposes a new way of classifying foods based on processing extent and purpose into three groups.*	NOVA, *original*
**Monteiro et al., 2010 (** **2** **)**	Brazil	(i), Methods development	*Provides an additional description of the original NOVA classification, introduced in 2009, based on processing extent and purpose, and apply it to Brazilian household food data as an example.*	NOVA, *original*
**Slimani et al., 2009 (** **39** **)**	Europe	(i), Cross-sectional	Describe the contribution of highly processed foods to total diet, nutrient intake, and dietary patterns across countries in Europe. *A new classification system based on food processing was developed to achieve this objective.*	EPIC, *Original*
**Asfaw, 2011 (** **40** **)**	Guatemala	(i), Cross-sectional	Explore the association between processed food consumption and body mass index in Guatemala. *A new classification system based on food processing was developed to achieve this objective.*	IFPRI, *original*
**Poti et al., 2015 (** **41** **)**	United States	(i), Open cohort	(1) Define a system to classify food products by the degree of industrial processing and separately by convenience; (2) Determine trends in energy contribution of processed and convenience food categories in purchases of consumer-packaged goods; (3) Compare the saturated fat, sugar, and sodium content of these purchases across different levels of processing and convenience.	UNC, *original*
**Byker Shanks et al., 2019 (** **42** **)**	United States	(i), Editorial	*Proposes a new food classification system based on food processing for the emergency food system, to limit consumption of UPFs.*	UP3, *original*
**Davidou et al., 2020 (** **43** **)**	France	(i), Methods development	(1) Enhance the NOVA classification by considering the ‘matrix’ effect, quantity of added salt, sugar, and fat, the degree of processing, and additives; (2) Refine the characterization and definition of UPFs; and (3) Propose a scoring system of foods to retailers and the agro-food industry; (4) evaluate packaged foods with this new classification system called Siga.	Siga, *original*
**Chajès et al., 2011 (** **45** **)**	Europe	(ii), Cross-sectional	(1) Investigate ecological-level associations between intakes of highly processed foods and plasma phospholipid elaidic acid levels; (2) Examine individual-level associations between plasma phospholipid elaidic acid levels and processed food intakes. *An updated version of the EPIC was used.*	EPIC, *updated*
**Monteiro et al., 2016 (** **3** **)**	Brazil	(ii), Commentary	*Proposes a new NOVA update, which categorizes foods into four groups based on processing extent and purpose, and a summary of its applications and findings to date.*	NOVA, *updated*
**Monteiro et al., 2017 (** **4** **)**	Brazil	(ii), Commentary	*Provides an additional detailed description of the updated 2016 NOVA classification, which categorizes foods into four groups based on processing extent and purpose.*	NOVA, *updated*
**Monteiro et al., 2019 (** **44** **)**	Brazil	(ii), Commentary	*Provides a guide and an additional detailed description of the updated 2016 NOVA classification, which categorizes foods into four groups based on processing extent and purpose.*	NOVA, *updated*
**Bleiweiss-Sande et al., 2020 (** **46** **)**	United States	(ii), Cross-sectional	(1) Adapt the current UNC classification system to include a home processing component; (2) Pilot test the adapted version using a nationally representative sample of foods consumed in the United States.	UNC, *Updated*
**Davidou et al., 2021 (** **47** **)**	France	(ii), Cross-sectional	Describe the markers of ultra-processing profile of UPFs in supermarkets. *An altered version of Siga was used in this study.*	Siga, *updated*

EPIC, system by European Prospective Investigation into Cancer and Nutrition (also known as IARC-EPIC or IARC); IFPRI, system by International Food Policy Research Institute; UNC, system by University of North Carolina; UP3, system by the Unprocessed Pantry Project.

a Articles are grouped as follows: (i) studies where a classification system was first introduced and detailed, (ii) studies describing an updated version of a classification system. Within each group, articles were sorted chronologically from oldest to newest.

b The country or broader geographical area where the study was conducted.

### Description of the included classification systems

Descriptions of both the original and updated versions of the six classification systems, NOVA, EPIC, IFPRI, UNC, UP3, and Siga, identified and included in this review are presented in Table 3. In the following section, the features of the most updated versions of the six systems are described, unless otherwise stated.

**Table 3:** Descriptions of the original and updated versions of the six identified classification systems

System, version, year	Categories and structure of classification system	Highly/ultra-processed food category ^a^			
		Definition	Food-processing techniques described	Additives described	Nutritional quality described
NOVA, *original (2009)* (1, 2)	1.0 Unprocessed and minimally processed foods 2.0 Processed culinary or food industry ingredients **3.0 Ultra-processed food products** ** 3.1 Ready-to-eat snacks or desserts** ** 3.2 Pre-prepared ready-to-heat products**	**3.0**. ‘Processing of a mix of Group 2 ingredients and Group 1 foodstuffs in order to create durable, accessible, convenient, and palatable ready-to-eat or to-heat food products liable to be consumed as snacks or desserts or to replace home-prepared dishes’.	Yes. Not part of core definition, but several techniques are described (e.g. salting, sugaring, baking).	Inconclusive. Not part of core definition. UPFs often-- but not always--contain preservatives, cosmetic additives, and synthetic micronutrients.	No
NOVA, *updated (2016)* (3, 4, 44)	1.0. Unprocessed and minimally processed foods 2.0 Processed culinary ingredients 3.0 Processed foods **4.0 Ultra-processed food products**	**4.0** ‘…industrial formulations typically with five or more and usually many ingredients. Such ingredients often include those also used in processed foods, such as sugar, oils, fats, salt, anti-oxidants, stabilisers, and preservatives. Ingredients only found in ultra-processed products include substances not commonly used in culinary preparations, and additives whose purpose is to imitate sensory qualities of Group 1 foods or of culinary preparations of these foods, or to disguise undesirable sensory qualities of the final product. Group 1 foods are a small proportion of or are even absent from ultra-processed products’.	Yes. Not part of core definition, but some techniques are described (e.g. extrusion and molding, and pre-processing).	Yes, groups of additives specific to UPFs are given, for example dyes, colors, flavors, emulsifiers.	No
EPIC, *original (2009)* (39)	1.0 Non-processed foods 2.0 Moderately processed foods 2.1 Industrial and commercial 2.2 Processed at home **3.0 Highly processed foods**	**3.0** ‘… industrially prepared, including those from bakeries and catering outlets, and which require no or minimal domestic preparation apart from heating and cooking’	Not part of definition, but examples of techniques listed in appendix.	No	No
EPIC, *updated (2011)* (45)	*1.0–2.0 as EPIC original.* 3.0 Processed foods 3.1 Processed staple/basic foods ** 3.2 Highly processed foods**	**3.2** ‘…industrially prepared and involving a high degree of processing such as drying, flaking, hydrogenation, heat treatment, use of industrial ingredients and industrial deep frying, including foods from bakeries and catering outlets, and which required no or minimal domestic preparation apart from heating and cooking…’ except ‘…processed staple/basic foods (e.g. bread, pasta, rice, milk, butter, vegetable oils)…’	Yes. Given as part of definition.	Not directly. Definition describes ‘industrial ingredients’.	No
IFPRI, *original (2011)* (40)	1.0 Unprocessed 2.0 Primary processed **3.0 Highly processed**	**3.0** ‘…have undergone secondary processing into a readily edible form’ **and** ‘… expected to contain high levels of added sugars, fat, and salt’.	No	No	Inconclusive. No cut-offs for added sugars, fat and salt.
UNC, *original (2015)* (41)	1.0 Unprocessed/minimally processed 2.0 Basic processed 2.1 Processed basic ingredients 2.2 Processed for basic preservation or precooking 3.0 Moderately processed 3.1 Moderately processed for flavor 3.2 Moderately processed grain product **4.0 Highly processed** ** 4.1 Highly processed ingredients** ** 4.2 Highly processed stand-alone**	**4.0** ‘Multi-ingredient industrially formulated mixtures processed to the extent that they are no longer recognizable as their original plant/animal source’ **4.1** ‘…consumed as additions’ ‘…typically consumed as condiments, dips, sauces, toppings, or ingredients in mixed dishes’. **4.2** ‘...typically not consumed as additions’	No	Not directly. Definition describes ‘multi-ingredient industrially formulated mixtures’.	No
UNC, *updated (2020)* (46)	*All categories as UNC original.* **4.0 Highly processed** ** 4.1 Highly processed ingredients** ** 4.2 Highly processed stand-alone**	*Same as UNC original except for additional criteria (home processing component (HP)) in categorizing mixed dishes:* *i) Foods prepared at home containing one or more highly processed culinary ingredients (4.1.) are categorized as moderately processed (3.0), not highly processed (4.0).* *ii) All foods that are breaded/battered/coated and deep fried are considered highly processed (4.0), regardless of whether they are homemade or not.*	No	Not directly. *As the original UNC.*	No
UP3, *original (2019)* (42)	1.0 Unprocessed food 1.1 Fresh 1.2 Pantry Staples 1.3 Prepared 1.3.1 Lightly 1.3.2 Heavily **2.0 Ultra-processed foods**	**2.0** ‘…has added artificial ingredients and uses cooking methods that are usually not found in home cooking. These ingredients include sweeteners and preservatives. This food is often high in added salt/sodium, sugar, saturated fat, unhealthy (hydrogenated) oils, and refined grains, and low in nutrients’.	No	Yes. Sweeteners and preservatives. Types not specified.	Inconclusive. Definition states these foods often (not always) have low nutritional quality.
Siga, *original (2020)* (43)	A0 Unprocessed foods A1 Minimally-processed foods (incl. culinary ingredients (A2)) B1 Nutritionally balanced processed foods B2 High salt, sugar, and/or fat level processed foods **C0.1 Nutritionally balanced ultra-processed foods level 0** **C0.2 High salt, sugar, and/or fat level** **Ultra-processed foods level 0** **C1 Ultra-processed foods**	‘characterized by the presence of at least one deliberately added substance obtained by synthesis or by a succession of physical, chemical and/or biological processes leading to its purification and/or substantial deterioration compared to the original material in the list of ingredients. UPFs can also be created by the direct application of a deterioration process (e.g. extrusion-cooking) to the food matrix. These substances are named MUPs and can be indifferently an ingredient or an additive, most of which are obtained by technological processes relating to cracking or synthesis’ **C0.1** Only one MUP1 and low levels ^b^ of salt, sugar, and/or fats **C0.2** Only one MUP1 and high levels ^b^ of salt, sugar, and/or fat **C1** At least one MUP2 and/or one at-risk additive ^c^	Yes, to some extent. Food processing techniques that destroy the food matrix of the original food such as ‘extrusion-cooking’.	Yes. MUPs, that are additives or ingredients added to foods, are part of the definition.	Yes. Part of definition for C0.1 and C0.2, based on the Food Standard Agency medium threshold ^b^
Siga, *updated (2021)* (47)	*A0–B2 as Siga original, except A1 and A2 merged into A1.* **C0.1 Nutritionally balanced ultra-processed foods level 0** **C0.2 High salt, sugar, and/or fat level** **Ultra-processed foods level 0** **C1 Ultra-processed foods level 1** **C2 Ultra-processed foods level 2** **C3 Ultra-processed foods level 3**	*The original Siga (2020) definition remains unchanged, except that sub-category C1 has been divided into three.* **C0.1:** *Only one MUP1 with low levels^b^ of salt, sugar, and/or fats* **C0.2**: *Only one MUP1 with high levels^b^ of salt, sugar, and/or fat* **C1** *2–5 MUP1* **C2** *> 5 MUP1 and/or 1 MUP2 and/or 1* at-risk additive^c^ **C3** *> 1 MUP2 and/or >1* at-risk additive^c^	Yes, to some extent. *As the original Siga*	Yes. *As the original Siga*	Yes. *As the original Siga*

UPFs, ultra-processed foods; MUP, marker of ultra processing; MUP1, marker of ultra processing obtained by chemical synthesis and is identical to natural substances, for example starch, natural flavoring; MUP2, marker of ultra-processing obtained by artificial chemical synthesis, for example artificial aroma.

a Systems with specific food processing examples are green, those with indirect descriptions are yellow, and those without descriptions are red. The table also shows if additives are included in the highly or ultra-processed food categories and if nutritional quality is considered, using the same color system.

b Based on the Food Standard Agency (FSA) medium threshold: 1.5 g salt/100 g, 12.5 g sugars/100 g, and 17.5 g fat/100 g for foods; and 0.75 g salt/100 g, 6.25 g sugars/100 g, and 8.75 g fat/100 g for beverages.

c At-risk additive, additives that have been noted to present a risk for health (safety concerns of the European Food Safety Authority reports, EFSA), for example sodium nitrite.

#### Overall structure of the systems

The number of categories in each system, including those for highly or UPFs, ranges from two to four main categories, with varying numbers of subcategories. All five systems share a similar first category, with a label spanning from ‘non’ and ‘un’-processed to minimally processed. However, the intermediate categories vary more in the naming, including ‘basic’, ‘primary’, ‘moderately’ processed, or ‘prepared’ food. For the last category or categories, NOVA, Siga, and UP3 use ‘ultra-processed’, while EPIC, IFPRI, and UNC use the term ‘highly processed’. The number of subcategories in the highly or ultra-processed category ranges from none in NOVA, UP3, EPIC, and IFPRI to two in UNC, which distinguishes between highly processed foods used as ingredients and those used as stand-alone foods. Siga, with five subcategories in the ultra-processed category, has the most subdivided UPF category.

#### Definitions of the highly or ultra-processed food categories

Definitions of the highly or ultra-processed food categories across the systems are given in Table 3. The length and detail given for the definition of the systems’ category describing the highly or UPF categories vary substantially between systems. There is a tendency that definitions in earlier systems are less detailed compared to later systems, which is evident in the updated versions of the systems. NOVA, EPIC, and Siga have, for instance, evolved to provide greater detail and distinctive criteria on what can be classified under the highly-processed food or UPF categories.

Most systems characterize these foods as products from industry, either explicitly (NOVA, EPIC, UNC, and Siga), using terms as ‘industrially’ prepared or ‘obtained by technological processes’, or indirectly, which is the case for the UP3 system that refers to ‘methods that are usually not found in home cooking’. The definition used by IFPRI is the most ambiguous, as it refers to foods in this category as having undergone ‘secondary processing into a readily edible form’. This definition may indirectly suggest that foods in this category are industrially produced, yet it does not clearly exclude homemade foods. Furthermore, in the UNC system, homemade foods that are breaded or battered or coated, and fried are classified under the highly processed category, irrespective of whether they were made at home or produced industrially.

#### Systems defining highly or ultra-processed foods considering specific food processing techniques

Although all identified systems consider food processing in their categorization, not all provide specific descriptions of food processing techniques in the highly or ultra-processed category. In Table 3, systems that give examples of specific food processing techniques are colored in green, those with indirect descriptions in yellow, and systems without specific descriptions are marked in red.

Three systems (NOVA, EPIC, and Siga) provide examples of specific food processing techniques in their highly or ultra-processed category definition. NOVA describes five techniques: ‘hydrogenation’, ‘hydrolysation’, ‘extrusion’, ‘moulding’, and ‘pre-processing for frying’. EPIC also lists five techniques: ‘drying’, ‘flaking’, ‘heat treatment’, ‘hydrogenation’, and ‘industrial deep frying’. In comparison, Siga focuses on the result of the processes in its definition, describing that UPFs can be made using food-processing techniques that destroy the food matrix of the original food, giving ‘extrusion-cooking’ as an example. The IFPRI, UNC, and UP3 do not provide examples of specific processing techniques for their highly or ultra-processed category.

#### Systems defining highly or ultra-processed foods considering additives

Table 3 also shows whether additives are included in the definition of highly processed food or UPF category, using the same green, yellow, and red color system as for food-processing techniques. Three systems (NOVA, Siga, and UP3) include additives explicitly in their categorization. NOVA describes categories of cosmetic additives specific to UPFs, such as colors, flavors, and emulsifiers. Siga is unique in directly applying substances called Markers of Ultra Processing (MUPs) that can be ingredients or additives, to categorize UPFs, subdividing the category based on the types and number of MUPs into subcategories C.01–C3, based on the potential health risks. The MUPs comprise three subgroups, MUP1, MUP2, and ‘At-risk additives’. MUP1s are described as substances that originate from chemical synthesis but remain identical to what is found naturally. Examples include starch or natural flavorings. MUP2s are synthesized artificially, such as artificial aromas. Siga defines the ‘At-risk additives’ to be additives that pose potential health risks, based on the European Food Safety Authority (EFSA) reports, an example being sodium nitrite. In contrast to Siga, the UP3 system describes sweeteners and preservatives without specifying which ones or any additional information. UNC references multi-ingredients as a characteristic of UPFs but does not clarify if these are additives. The EPIC mentions industrial ingredients without providing examples of specific additives, while IFPRI does not include additives in its definitions.

#### Systems defining highly or ultra-processed foods considering nutritional quality

Whether the systems account for nutritional quality in their highly processed food or UPF category is also shown in Table 3, using the same color system as for food-processing techniques and additives. Siga is the only system that includes aspects of nutritional quality using defined cut-offs for sugar, fat, and salt content in specific subcategories (C0.1 and C0.2). The C0.1 category is for nutritionally balanced UPFs, with cut-offs based on thresholds set by the Food Standard Agency (FSA), whereas the C0.2 in Siga is above this threshold. Siga’s remaining categories for UPFs (C1–C3) focus on the presence of MUPs, without considerations regarding nutritional quality. IFPRI and UP3 are less explicit about nutritional quality, suggesting that UPFs in their systems are ‘expected to contain’/‘often’ high in added sugars, fat, and salt, and for UP3, this also includes high in refined grains and low in nutrients. NOVA, EPIC, and UNC do not include nutritional quality as part of their UPF category definitions.

## Discussion

In this systematic review, we identified six systems, which categorize highly or UPFs: NOVA, EPIC, IFPRI, UNC, UP3, and Siga. These systems vary in structure and definitions, with later systems being more detailed. All systems consider food processing in their categorization and define highly processed food or UPFs as industrial products, either directly or indirectly. NOVA, EPIC, and Siga provide specific examples of food-processing techniques. For additives, NOVA, Siga, and UP3 include them explicitly, with Siga being the most detailed, categorizing UPFs based on types of additives and other ingredients (MUPs). Siga also uniquely includes quantitative measures for nutritional quality, using defined cut-offs for sugar, fat, and salt, while IFPRI and UP3 address nutritional quality in a non-quantitative way.

In the following, we will compare the highly processed food or UPF categories of the systems identified in this review. Specifically, we will focus on the specificity of food-processing techniques, additives, and nutritional quality aspects in the systems’ UPF definitions. Finally, we will explore the usefulness and practical applications of these UPF definitions.

### Food processing techniques – comparison of the six identified systems

The highly or UPFs categories of all the six systems identified in this review refer to industrial food-processing techniques, either directly or indirectly. NOVA’s updated definition from 2016 describes these foods as made using several different successive food processing techniques not used in home kitchens, suggesting that these are industrial processes only (3, 4). This is in line with the other systems in this review, except the updated UNC system (46), which also includes some homemade foods.

NOVA further provides specific examples of food-processing techniques exclusive for NOVA UPFs, with no domestic equivalents (3, 4, 44). Five examples are given as part of its UPF definition, which includes hydrogenation and hydrolyzation, extrusion and molding, and pre-processing for frying (4). EPIC (45) and the Siga system (47) also provide some specific examples in their UPF category but do not offer a finite list of processing techniques. Many food-processing methods, like drying and high-temperature treatment, offer benefits such as microbiological safety and making foods more appealing (48). However, they can also lead to vitamin loss and harmful process contaminants, including acrylamide and heterocyclic aromatic amines, which may pose health risks (48, 49). Process contaminants, such as acrylamide, can form in both home kitchens and industrial settings during heat treatments (48). Current UPF definitions in systems identified in this review do not adequately address this issue, as they all fail to account for process contaminants resulting from processing techniques beyond those specific to the UPF category. That is, none of the existing UPF definitions is specific for process contaminants, regardless of providing specific examples of processing techniques (NOVA, EPIC, and Siga) or not.

Some food-processing techniques can deteriorate the food matrix and may affect absorption of nutrients and the feeling of satiety (10, 50). NOVA, UNC, and Siga focus on the food matrix in their UPF categories. NOVA and UNC define UPFs as foods with a destroyed food matrix, whereas Siga also refers to processes related to the food matrix. NOVA describes UPFs as ‘formulations made mostly or entirely from substances derived from foods and additives, with little if any intact Group 1 food’ (4). UNC defines them ‘multi-ingredient industrially formulated mixtures processed to the extent that they are no longer recognizable as their original plant/animal source’ (46). Siga mentions ‘substantial deterioration compared to the original material in the ingredient list’, even though Siga uses primarily MUPs, not processing techniques, to define UPFs (47). In comparison, EPIC, IFPRI, and UP3 lack clear references to the food matrix.

Despite NOVA, UNC, and Siga classifications focus on alterations of the food matrix, not all provide specific examples of processing techniques exclusive to the UPF category that lead to dietary reconstitution. While UNC does not offer any examples, both NOVA and Siga mention techniques like extrusion, which significantly deteriorates the food matrix. Extrusion involves processing raw ingredients through a restricted opening using a rotating helical screw under high temperature and pressure, producing foods like snacks and breakfast cereals (51). However, not all of NOVA’s examples of UPF-specific food-processing techniques, such as pre-processing for frying (52), degrade the food matrix to the same extent as extrusion. This conflicts with NOVA’s core definition, which states that UPFs have ‘little if any intact Group 1 food’ (4), raising questions about its validity for foods pre-processed for frying, like fish fingers. The UNC system (46) presents an even larger conflict between its core definition and the example of defining homemade foods, like battered and deep-fried fresh fish fillets, as highly processed, where the fish remains recognizable.

In evaluating food-processing techniques in UPF definitions, including homemade foods seems like a distraction. The UNC classification system (46) includes deep-fried foods, whether homemade or not. While this could guide consumers toward healthier diets, it may complicate the clarity and usefulness of UPF categorization. Nevertheless, if one wants to focus on process contaminants, homemade foods could have a place. However, from a food matrix perspective, homemade foods are less important, as processes leading to dietary reconstitution, such as extrusion or hydrolyzation, are typically exclusive to industrially produced foods (51, 53).

None of the UPF definitions of the systems identified in this review is clear and specific about food-processing techniques. The lack of mandatory food labeling on food-processing techniques adds to this problem, making it impossible for consumers to know what specific processing techniques their food has undergone. Without a comprehensive list of processing techniques exclusive to UPFs, using this specific aspect to define UPFs is neither feasible nor a valid criterion for categorizing them. However, since MUPs (additives and ingredients) result from many processes specific to UPFs (54), we argue that these markers, rather than specific food-processing techniques, should be used to classify UPFs.

### Additives – comparison of the six identified systems

Three of the identified systems, NOVA, Siga, and UP3, explicitly include additives in their UPF classifications (3, 42, 47), while the others do not. The depth of detail about additives varies among these systems. UP3 focuses on sweeteners and preservatives only (42), while NOVA describes markers exclusive to UPFs (4). According to NOVA, UPFs must contain at least one marker of ultra-processing, such as unconventional energy sources (like casein, lactose, hydrolyzed proteins, and high-fructose corn syrup) or additives that improve sensory qualities (e.g. colors, flavors, non-sugar sweeteners, and various processing aids such as emulsifiers) (4, 44). By doing so, NOVA emphasizes the purpose and function of ingredients and additives rather than their potential health effects, advocating for reducing non-essential ones. Siga further specifies the number and types of ingredients and additives unique to UPFs, including MUP1/2 and at-risk additives within specific UPF subcategories (47). Although this introduces an additional level of complexity, Siga acknowledges the diverse nature of additives.

Among the three systems including additives in their UPF definition, NOVA and Siga are specific and clear, detailing ingredients and additives exclusive to their UPF categories. The next question is whether additives can be used as a valid criterion for categorizing UPFs, and if it is, whether NOVA or Siga provides a better approach.

In the EU, around 300 food additives are approved (55), with slightly more allowed in the US (56). While EFSA considers approved additives safe at specified levels, not everyone agrees. For example, despite being reevaluated and deemed safe by EFSA in 2017 (57), nitrates and nitrites are linked to increased colorectal cancer risk according to the French Agency for Food, Environmental and Occupational Health & Safety (ANSES). Consequently, ANSES recommended minimizing their use in 2022 (58). Siga classifies these as at-risk additives, placing them in the lowest UPF subcategory. In the NOVA system, nitrates and nitrites are likely defined as additives exclusive to the UPFs, as they are multifunctional, serving not only as preservatives but also enhancing the color and flavor of foods.

Another example of a potentially harmful substance used for cosmetic purposes is the red food color Allura Red AC. It is approved in the EU (59), and in the US, it is approved under the condition that foods containing it are labeled ‘may have an adverse effect on activity and attention in children’ (56). Ingredients like the protein casein, which is associated with positive health claims (60), are regarded by NOVA as equally problematic as nitrates and Allura Red AC, as they are makers specific to UPFs. In contrast, Siga distinguishes between MUP1s like casein that are natural substances, and other MUPs, including at-risk additives.

Compared to food-processing techniques, the parts of the UPF definitions related to additives are clearer and more coherent. While UP3 is vague, both NOVA and Siga specify ingredients and additives exclusive to their UPF categories. By adopting the precautionary principle regarding additives, acknowledging that not all are necessary substances in food, and that they have diverse risks, Siga is preferable to NOVA.

### Nutritional quality – comparison of the six identified systems

Across all identified systems, nutritional quality aspects are either lacking or vague, except for Siga. NOVA’s UPF definition has been criticized for not considering nutritional quality (61). Its foods ‘typically’ include sugars, oils, fats, and salt, but these ingredients are not exclusive to the NOVA category four (4). Furthermore, no cut-offs or specifications of are given, similar to IFPRI (40) and UP3 (42). In contrast, Siga uniquely and quantitatively considers nutrient content in two of its UPF subcategories. This means more favorable foods, with low levels of sugar, fat, or salt, have a lower degree of ultra-processing, if they contain maximum one MUP1, a marker of ultra-processing identical to natural substances, like natural flavoring. This responds directly to the criticism of NOVA (43), and other current classification systems for UPFs, not taking nutritional quality into account. While NOVA combined with NutriScore (62) or similar could separate nutritional quality from other UPF aspects, and because consumers have shown they can independently identify and understand these complementary dimensions of foods (63), this could also respond to the criticism. However, the significant critique from parts of the scientific community suggests that nutritional quality should be part of a meaningful UPF definition to reach scientific consensus. Of the systems identified in this review, Siga is currently the only one with specific and useful criteria for aspects of nutritional quality as part of its UPF definition.

### Usefulness and practical application of the UPF definitions

There is an ongoing debate and lack of consensus within the scientific community about the concept of UPFs. This is apparent from how UPFs (as defined by NOVA) has been criticized by nutrition bodies like the British Nutrition Foundation (61) and Nordic Nutrition Recommendations from 2023 (64) for not being useful, as it groups both energy-dense and micronutrient-poor foods together with healthier UPFs, such as whole grain bread and sugar-sweetened soft drinks, into the same category (61). Despite this, several countries, such as Brazil, have integrated the NOVA framework into their dietary guidelines (65).

In this review, available systems were identified and evaluated to explore alternatives that might address the criticisms directed at NOVA. Among the systems reviewed, NOVA and Siga stand out for their specificity and clarity in identifying additives unique to the UPF category, setting them apart from other systems like EPIC, IFPRI, UNC, and UP3. Following this, a key question is whether Siga’s detailed, reductionistic approach is superior to NOVA’s or for whom.

For consumers, it seems impossible to classify foods using Siga, involving MUP1/2 and at-risk additives. A study using the Siga classification identified 92 MUP1 and 60 MUP2 in packaged UPFs (47), demonstrating its complexity. According to Moubarac et al., simpler systems like NOVA make communicating and implementing dietary recommendations easier (22). In any case, if mandatory labeling for UPFs is implemented, system complexity becomes less critical, as consumers would not need to grasp the underlying concepts.

There is no consensus on the best approach for policymakers to regulate and control the industry. Some argue that having several UPF subgroups, like Siga, could benefit the food industry. By including nutritional quality as a maker, producers may ‘green wash’ their products by adding more nutrients, which distracts from issues related to the degradation of the food matrix and MUPs, which make UPFs different from less-processed foods (54, 66). Others argue that the broad UPF category in the NOVA classification hinders the food industry’s efforts to reformulate products to be more nutritious (67). Furthermore, at a November 2024 London conference on UPFs, WHO was noted to be working on a new operational UPF definition, and it was advocated for integrating UPF definitions into existing High in Fat, Salt and Sugar (HFSS) regulations (68, 69).

A reductionistic approach like Siga’s appeals to researchers trying to disentangle the various factors linking UPFs to negative health outcomes. However, both Siga and NOVA share the same underpinning methodological challenges, related to the lack of sufficiently detailed dietary information from epidemiological studies to accurately classify these foods (70, 71). This remains an unresolved issue moving forward.

### Strengths and limitations

A key strength of this systematic review is its comprehensive search across multiple databases. Blind screening with Rayyan, with at least two researchers independently reviewing each study, was used to reduce bias and enhance objectivity. A limitation is the restriction to English-language articles, which may exclude relevant classification systems published in other languages, and the exclusion of grey literature.

## Conclusion

In this systematic review, we identified six distinct food classification systems, which include a category for highly processed food or UPFs: NOVA, EPIC, IFPRI, UNC, UP3, and Siga. None of these systems provide clear and specific definitions for food-processing techniques. However, in terms of additives, both NOVA and Siga specify ingredients and additives unique to their UPF categories with clear definitions. Siga further distinguishes itself by recognizing the diverse risks associated with additives and offers specific criteria for nutritional quality within its UPF definition, addressing calls to incorporate nutritional quality into such definitions. Among the systems reviewed, Siga addresses several criticisms aimed at the currently much used NOVA. However, Siga does not offer enhancements and clarity over NOVA concerning food-processing techniques related to the food matrix. In developing a new operational definition for UPFs, WHO may consider Siga as a valuable, but not final step, forward.

## Supplementary Material


