# Supplementary material for: Definitions of ultra-processed foods beyond NOVA: a systematic review and evaluation
Source: Food Nutr Res. 2025 Jun 16;69:10.29219/fnr.v69.12217. doi: 10.29219/fnr.v69.12217 (PMC12255158; doi:10.29219/fnr.v69.12217)
Supplement: Supplementary file 1 [file FNR-69-12217-s1.docx]

Supplemental Table S1: Main characteristics of 15 articles excluded at final exclusion step ordered chronologically by group ^a^

| Authors, year | Group ^a^, Study type | Objective(s)/  description of classification system(s) in study if not clear from objective(s) | Study setting |
| --- | --- | --- | --- |
| Moubarac et al., 2014 (1) | (I), Systematic review | Conduct a systematic review of food classification systems that include processing; assess and score them on how specific, coherent, clear, comprehensive, and workable they are; and describe their relevance to food, nutrition, health and use in various settings. *This study describes five classification systems: EPIC (IARC-EPIC), IFIC, IFPRI, NIPH and NOVA* | Brazil **^b^** |
| Knorr & Watzke, 2019 (2) | (I), Narrative review | *This paper describes and discusses the role of food processing in history, new technologies in relation to nutrition and health, and the usefulness of the NOVA classification system.* | Germany ^b^ |
| Sadler et al., 2021 (3) | (I),  Critical literature review | Critically evaluate processed food classification systems’ constructs by qualitatively examining definitions and underlying intentions, evaluating findings from diverse perspectives, and highlighting key areas of debate. *This study describes eight classification systems: EPIC (IARC-EPIC), IFIC, IFPRI, Louzada (variation of NOVA), NIPH, NOVA, Siga and UNC (Poti).* | United Kingdom ^b^ |
| Gibney et al., 2022 (4) | (I),  Narrative review | *This study reviews the comparability of four existing classification systems (EPIC, IFIC, NOVA, UNC) and addresses the challenges and gaps in research between the health and processing of foods.* | Ireland ^b^ |
| Jideani et al., 2022 (5) | (I),  Narrative review | *This study briefly discusses existing food processing classification systems, which category some African foods fall into, challenges, and future directions. In total, eight classification systems are described: EPIC (IARC-EPIC), FSANZ, IFIC, IFPRI, NIPH, NOVA, Siga and UNC (Poti).* | South-Africa ^b^ |
| Forde, 2023 (6) | (I),  Narrative review | Identify key considerations and gaps in current research that can guide future studies on the impact of processed foods on health. *This study focuses on the NOVA classification system.* | Netherlands ^b^ |
| Bleiweiss-Sande et al., 2019 (7) | (II), Comparative study | Compare inter-rater reliability, food processing category, and the relationship between processing category and nutrient concentration across three food processing classification systems *(IFIC, NOVA, and UNC).* | United States ^c^ |
| Martinez-Perez et al., 2021 (8) | (II), Comparative study | Evaluate how different food classification systems (EPIC, IFIC, NOVA, UNC) affects the cross-sectional relationship between UPFs consumption and cardiometabolic health. | Spain ^c^ |
| de Araújo et al., 2022 (9) | (II), Comparative study | Compare five food classification systems based on processing (EPIC, IFIC, IFPRI, NOVA, UNC) and assess their comparability in evaluating the prevalence of high/UPFs in the overall diet. | Portugal ^c^ |
| Gibney, 2019 (10) | (III), Opinion paper | Examines the evolution of the definition of UPFs since its introduction. *The NOVA is the focus, but EPIC is also discussed.* | Ireland ^b^ |
| Blondin et al. 2021 (11) | (III), Intervention | (1) Categorize foods children bring from home to school by processing level; (2) Assess if a school-based intervention effectively reduces the proportion of energy from highly processed foods brought from home. *The UNC system was used in this study.* | United States ^c^ |
| Larison et al., 2021 (12) | (III), Case study | Investigate the impact of the early phase of the COVID-19 pandemic on the food supply of two food pantries. *The NOVA and UP3 were used in this study.* | United States ^c^ |
| Botelho et al., 2022 (13) | (III), Methods development | Outline the development and revision of a food categorization tool based on the extent and purpose of industrial processing (NOVA) and evaluate its practical application. | Brazil **^b^** |
| Ebner et al., 2022 (14) | (III), Cross-sectional | (1) Examine the relationship between level of food processing and nutrition quality for packaged foods; (2) Identify which markers of ultra-processing may modify foods’ compositional scores. *The Siga was used in this study.* | France ^c^ |
| Zancheta Ricardo et al., 2023 (15) | (III), Cross-sectional | Assess how often UPFs appeared in the diets and beverages of Chilean preschoolers, utilizing three distinct methods to identify UPFs based on the NOVA classification system. | Chile ^c^ |

EPIC, system by European Prospective Investigation into Cancer and Nutrition (also known as IARC-EPIC or IARC); IFIC, system by International Food Information Council; IFPRI, system by International Food Policy Research Institute; NIPH, system by National Institute of Public Health in Mexico; UNC, system by University of North Carolina, FSANZ, system by Food Standards Australian New Zealand.

^a^ Articles are grouped as follows: (I) reviews of existing classification systems, (II) comparative studies of existing classification systems, and (III) other studies describing existing classification systems, or their use. Within each group, articles were sorted chronologically from oldest to newest.

^b^ The country of affiliation of the first author.

^c^ The country or broader geographical area where the study was conducted.

References for Supplemental Table S1

1. Moubarac J-C, Parra DC, Cannon G *et al.* (2014) Food Classification Systems Based on Food Processing: Significance and Implications for Policies and Actions: A Systematic Literature Review and Assessment. *Current obesity reports* 3, 256-272.
2. Knorr D, Watzke H. Food Processing at a Crossroad. *Front Nutr*. 2019;6:85.
3. Sadler CR, Grassby T, Hart K *et al.* (2021) Processed food classification: Conceptualisation and challenges. *Trends in Food Science & Technology* 112, 149-162.
4. Gibney MJ & Forde CG (2022) Nutrition research challenges for processed food and health. *Nat Food* 3, 104-109.
5. Jideani AIO, Onipe OO Ramashia SE (2022) Classification of African Native Plant Foods Based on Their Processing Levels. *Frontiers in nutrition* 9, 825690.
6. Forde CG. Beyond ultra-processed: considering the future role of food processing in human health. *Proc Nutr Soc.* 2023;82(3):406-18.
7. Bleiweiss-Sande R, Chui K, Evans EW *et al.* (2019) Robustness of Food Processing Classification Systems. *Nutrients* 11.
8. Martinez-Perez C, San-Cristobal R, Guallar-Castillon P *et al.* (2021) Use of Different Food Classification Systems to Assess the Association between Ultra-Processed Food Consumption and Cardiometabolic Health in an Elderly Population with Metabolic Syndrome (PREDIMED-Plus Cohort). *Nutrients* 13.
9. de Araújo TP, de Moraes MM, Afonso C *et al.* (2022) Food Processing: Comparison of Different Food Classification Systems. *Nutrients* 14.
10. Gibney MJ. Ultra-processed foods: definitions and policy issues. *Curr Dev Nutr*. 2019;3(2):nzy077
11. Blondin SA, AlSukait R, Bleiweiss-Sande R *et al.* (2021) Processed and Packed: How Refined Are the Foods That Children Bring to School for Snack and Lunch? *J Acad Nutr Diet* 121, 883-894.
12. Larison L, Byker Shanks C, Webber E *et al.* (2021) The Influence of the COVID-19 Pandemic on the Food Supply in the Emergency Food System: A Case Study at 2 Food Pantries. *Curr Dev Nutr* 5, nzab115.
13. Botelho AMC, A.M.; Mazzonetto, A.C.; Fiates, G.M.R. Decision flowchart for food classification by the extent and purpose of industrial processing: update and practical application. Rev Nutr. 2022;35.
14. Ebner P, Frank K, Christodoulou A *et al.* (2022) How are the processing and nutrient dimensions of foods interconnected? an issue of hierarchy based on three different food scores. *Int J Food Sci Nutr* 73, 770-785.
15. Zancheta Ricardo C, Duran AC, Grilo MF, Rebolledo N, Díaz-Torrente X, Reyes M, et al. Impact of the use of food ingredients and additives on the estimation of ultra-processed foods and beverages. *Front Nutr.* 2022;9:1046463.
